# Theoretical Study of CH_4_ and CO_2_ Separation by IRMOFs

**DOI:** 10.1021/acsomega.4c04482

**Published:** 2024-09-04

**Authors:** Ana Luiza Andrade Mizuno, Edna da Silva Machado, João B. L. Martins, José Roberto dos Santos Politi, Nailton Martins Rodrigues

**Affiliations:** † Instituto de Química, Universidade de Brasília, 70910-900 Brasília, DF, Brasil; ‡ Departamento de Química, Universidade Federal do Maranhão, 65085-580 São Luís, MA, Brasil

## Abstract

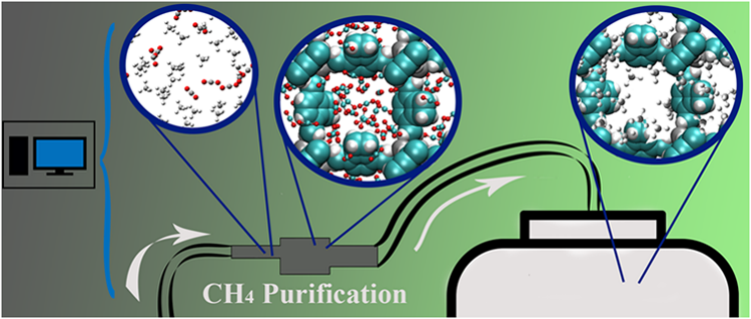

Porous materials such as isoreticular metal–organic frameworks (IRMOFs) can be applied in several areas that explore the physical adsorption. An area that has gained prominence is fuel gas storage, as it provides the storage of a large amount of gas at low pressure and the purification of combustible gas due to the selectivity of the different chemical environments of its pores. IRMOFs represent an ideal study group due to their wide range of pore sizes resulting from the use of different organic ligands. In this context, exploring IRMOFs that adsorb more efficiently stands out, mainly for optimizing the ligand, pressure, and temperature. This work focused on the adsorption and separation of CH_4_ and CO_2_ using various IRMOFs. The results suggest that IRMOF-6 is the most suitable for separation and purification and that enhanced purification occurs when the temperature is reduced and the system pressure is increased. This better performance is associated with the higher adsorption energies for this MOF, with CO_2_ being higher than CH_4_, which tends to become even more evident when the system pressure increases.

## 1 Introduction

Natural gas is an energy source that is increasingly used, with methane being the main component of its composition.^1^ The discovery of new natural gas reserves has continuously increased in recent decades, making its price more affordable, usually lower than gasoline.^2^ Several applications have become viable and attractive, such as the use of natural gas in motor vehicles. Fuel gas storage in motor vehicles is carried out using high-pressure cylinders, which creates a high risk of explosion,^3^ and a way to prevent this problem is by using cylinders with porous materials to store the fuel.^4^

The adsorption capacity of the porous material makes it possible to store a more significant amount of gas at lower pressures, reducing the risk of explosions. In this context, using metal–organic frameworks (MOFs) as porous material has been gaining prominence.^5−7^ MOFs are made up of an inorganic unit and an organic unit, the first of which has a greater efficiency in adsorption. This combination allows the formation of structures with high regular porosity, high surface area, low density, and considerable thermal and chemical stability, and these properties lead to a large number of applications^8−12^ in different fields, such as biomedicine,^13,14^ photonics,^15,16^ catalysis,^17,18^ gas storage and separation,^19−22^ and others.^23−25^ Between the adsorption sites of the structure, the inorganic site generally adsorbs molecules most efficiently.^26,27^

The composition of natural gas is approximately 95% methane and traces of ethane, propane, and contaminants such as H_2_S and CO_2_, the latter two of which, in addition to being toxic and reducing the combustion power of natural gas, can quickly saturate inorganic MOF units and reduce the gas storage capacity in cylinders containing porous materials. The previous removal of these contaminants is crucial for use in motor vehicles. In this context, MOFs again appear promising materials for being applied in separating these pollutants and consequent natural gas purification^28^ for later storage and use.

In addition to their role in gas storage and separation, MOFs also hold promise in addressing environmental concerns. Carbon dioxide (CO_2_), one of the main contributors, is also one of the agents responsible for the greenhouse effect and can be adsorbed on MOFs and converted into less harmful molecules to the environment. This photocatalytic transformation of CO_2_ into small organic molecules, such as methanol (CH_3_OH), makes MOFs highly recommended for this application.^29^

Yaghi et al. were pioneers in the evaluation of MOFs for gas adsorption, as well as presented new structures, with emphasis on a compound containing Zn(II) and 1,4-benzenedicarboxylate known as MOF-5.^30^ The MOF-5 (or IRMOF-1) is one of the most studied structures, both experimentally and theoretically,^31^ and gave rise to a new class of MOFs named isoreticular MOF (IRMOF). Yaghi’s group^32^ also evaluated the CH_4_ storage capacity in the 15 new structures (IRMOFs) and found that among these structures, IRMOF-6 was the one that showed the best results, a feature attributed to its large surface area and pore volume.

When evaluating the potential of IRMOF-1 in the adsorption of several gases, including CH_4_, Snurr et al. concluded using Grand Canonical Monte Carlo (GCMC) simulations that the molecules tend to adsorb preferentially on the inorganic subunit, with the preferred position of interaction being on the oxygens^33^ coordinated to the metallic centers and forming the Zn_4_O set.

Studying the use of IRMOF-1 in CO_2_ capture, Sarmiento-Perez et al.,^34^ using GCMC calculations, showed that the aromatic ring of the ligand 1,4-benzene-dicarboxylate (BDC) interacts with CO_2_, but this occurs to a lesser extent when compared to the inorganic part. Moreover, using computer simulation, Li et al. studied the performance of 151 MOFs in the adsorption and desorption of CO_2_ and CH_4_ at increasing temperatures, establishing that temperatures between 323 and 423 K are sufficient to promote the desorption of both gases.^35^ Simulations of the vapor–liquid equilibrium (VLE) are computationally expensive. However, the parameter of the GenericMOF force field contained in the RASPA program was widely used to study the solubility of CO_2_ and other small molecules, where the properties are computed with the canonical Gibbs ensemble.^36−39^ For the purpose of this work, GCMC was used to compute the adsorption properties for the gas phase adsorption of CH_4_ and CO_2_. In this way, molecular simulations were consistently addressed to the study of vapor–liquid equilibria applying force field models in the simulations, and the behavior of these parameters to fit experimental data was recently discussed.^40−43^

The use of computational tools for these studies has promoted obtaining beneficial information,^44^ with the potential to leverage significant advances in this area. From this perspective, we investigated the potential of IRMOF-1, 6, 8, 10, 14, and 16 to adsorb CH_4_ and CO_2_ and for gas separation. This study used density functional theory (DFT), the semiempirical method, and GCMC simulations.

## 2 Methodology

All GCMC simulations were carried out using the RASPA program^36^ and performed at 1–80 bar and 298 K to simulate the same standard conditions application.^45^ The unit cell for each MOF was used, and the structures of IRMOF-1, 6, 8, 10, 14, and 16 were obtained from the literature.^32^ The CH_4_ molecule was described with a single-sphere model, and all parameters used for MOFs and gas molecules can be seen in the Supporting Information. The parameter describing the interaction between the MOF and gas molecules was calculated using Lorentz–Berthelot (eqs 1 and 2) mixing rules from Lennard-Jones potential parameters.
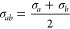
1
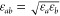
2The atomic charges of MOFs were calculated using the charge-equilibration scheme of Snurr,^46^ while for CO_2_, CHelpG charges was used, calculated with B3LYP/6-311++G** in the Gaussian09 program.^47^ The Coulombic interactions were obtained using the Ewald method.^48,49^ These simulations generated helium fractions, adsorption values, potential maps, radial distributions, average interaction energies, and contributions to the interaction energy.

The interaction energy between two gas molecules was determined from the potential energy curve calculated using the semiempirical PM6^50^ method with D3 correction for dispersion^51^ on MOPAC2016^52^ and the isocontour generated with iRASPA.^53^

## 3 Results and Discussion

### 3.1 IRMOF Parameters

Table 1 shows the cell parameters (*a*, *b*, *c*, α, β, and γ). The cell volume was calculated from the cell parameters. The pore volume was obtained from the RASPA program, along with the percentage of pore volume relative to cell volume (% pore) of each IRMOF. All structures were obtained from RASPA program,^36^ which references the work of Yaghi’s group.^32^

**Table 1:** Cell Parameters, Volume of Unit Cell, Pore Volume, and Pore Volume Percentage for the IRMOF-1, 6, 8, 10, 14, and 16

	cell parameters						volume (Å^3^)		
MOF	*a*	*b*	*c*	α	β	γ	cell	pore	% pore
IRMOF-1	26.832	26.832	26.832	90.0	90.0	90.0	19,317.86	15,536.36	80.42
IRMOF-6	25.842	25.842	25.842	90.0	90.0	90.0	17,257.19	13,276.53	76.93
IRMOF-8	30.092	30.092	30.092	90.0	90.0	90.0	27,249.16	22,795.26	83.65
IRMOF-10	34.281	34.281	34.281	90.0	90.0	90.0	40,286.58	35,622.28	88.42
IRMOF-14	34.381	34.381	34.381	90.0	90.0	90.0	40,640.17	37,410.29	92.05
IRMOF-16	42.980	42.980	42.980	90.0	90.0	90.0	79,396.11	72,492.14	91.30

### 3.2 Pure Component: CH_4_

**Figure 1:**
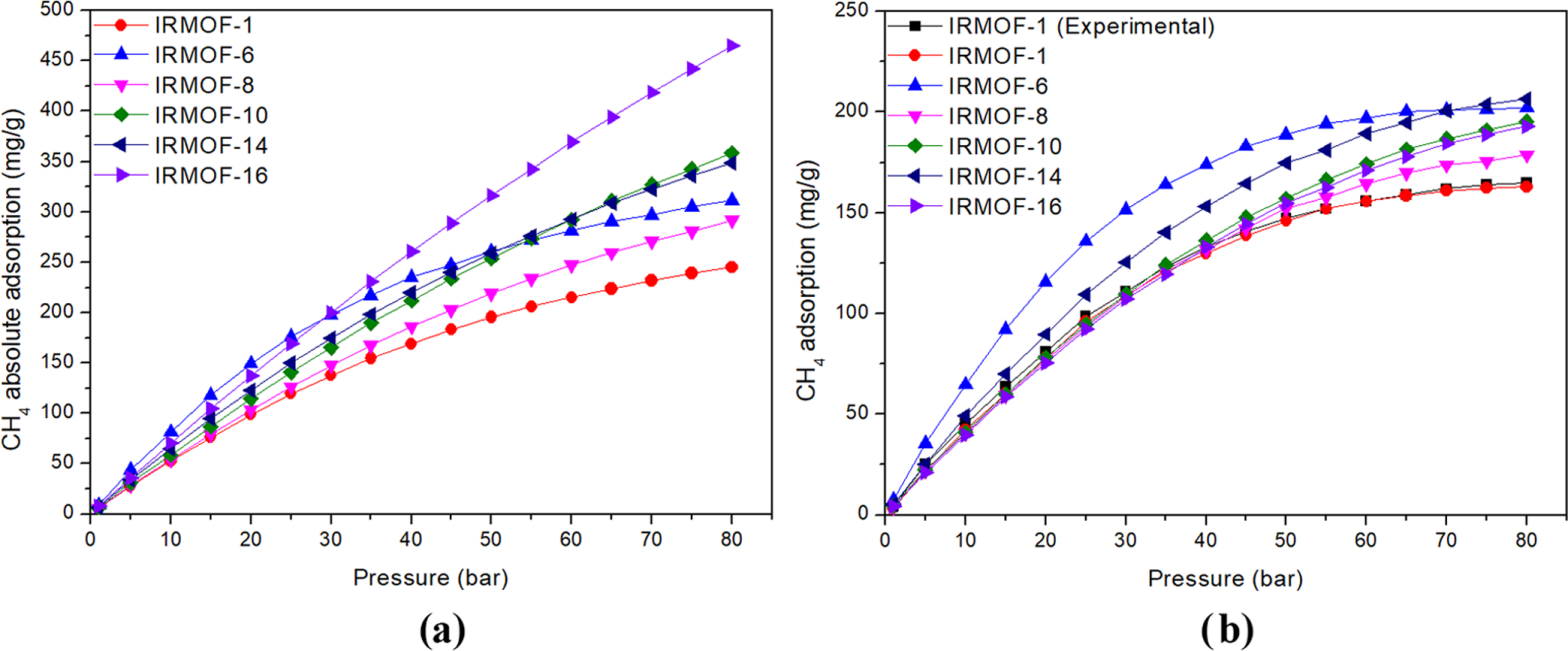
Absolute adsorption isotherms (a) and the excess adsorption isotherms (b) in mg/g for CH_4_ for the IRMOFs studied.

The absolute adsorption isotherms (Figure 1a) and excess adsorption isotherms (Figure 1b) were obtained from the crystallographic structures of each IRMOF. The absolute adsorption isotherm accounts for all gas molecules, even those that are there, simply because there is free space to be occupied. In contrast, the excess adsorption isotherm eliminates these molecules and only considers those that adsorb. In this context, in applications where adsorption is a relevant factor, such as applications involving low pressure, it is more advisable to work with excess adsorption isotherms, which will be followed throughout the manuscript. Applications requiring high pressures need a simulation under conditions different from the modeling in the present study.

For the absolute adsorption isotherms, the increase in pressure triggers IRMOFs with the smallest pore volumes to start filling the empty spaces, while those with larger pores will saturate only at higher pressures. Therefore, at high pressures, the absolute adsorption becomes a data directly proportional to the pore volume (as will be seen later), so that IRMOF-1 will have the lowest molecule storage capacity and IRMOF-16 will have the highest storage capacity. This trend has significant implications for the design and optimization of these materials, involving gas uptake.

For the excess adsorption isotherm, the results showed agreement between experimental data^5^ and those calculated for the adsorption isotherm of IRMOF-1 at 298 K. Among the structures, IRMOF-6 showed the best adsorption ratio in mg/g up to 65 bar. Above this pressure, IRMOF-14 had the best performance.

**Figure 2:**
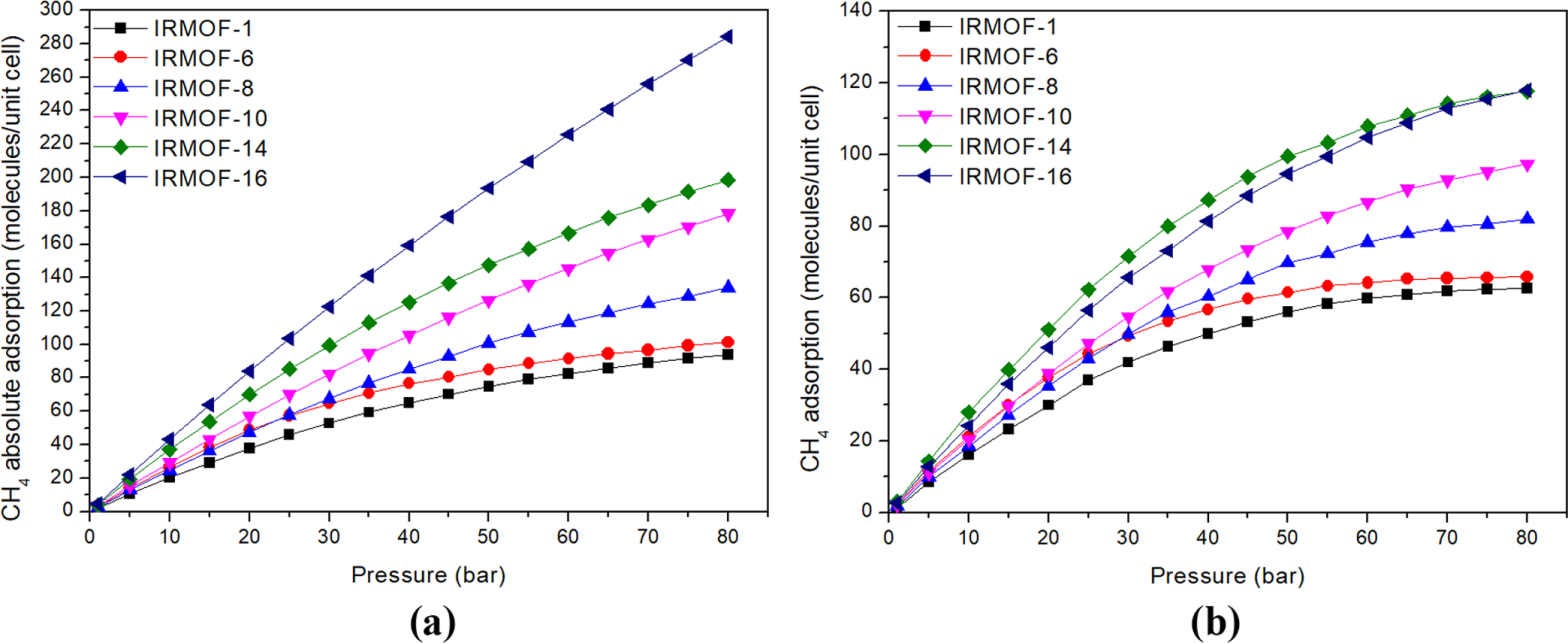
Absolute adsorption isotherms (a) and the excess adsorption isotherms (b) in molecules/unit cell for CH_4_ in the different structures studied.

Although the adsorption isotherms given in mg/g are relevant data, a parallel between the adsorption capacity and the pore structures can be better explored using adsorption isotherms in molecules/unit cell (Figure 2).

The absolute adsorption isotherm depicted in Figure 2a shows that the higher the pressure, the greater the number of molecules in the pore of each MOF, taking into account the adsorbed molecules and those just occupying empty spaces. Therefore, the relationship between the pore volume and the number of molecules contained therein is the most significant for higher pressures. IRMOF-16 presents better performance due to its larger pore volume. However, Figure 2b suggests a direct relationship with physical adsorption; when this is taken into account, the scenario changes. Throughout the text, all information about isotherms is based on data referring to the excess adsorption isotherm.

For excess adsorption isotherms data (Figure 2b), it is possible to observe that up to 75 bar, the IRMOF-14 is the one that adsorbs the most significant number of molecules per unit cell. As from 80 bar, the IRMOF-16 is the one that starts to have the highest adsorption capacity, which is slightly larger than that of the IRMOF-14, so at this pressure, there is a direct relationship between adsorption capacity and pore volume. This relation becomes more evident, observing that the adsorption in Figure 2 directly follows the pore volume percentage (% pore in Table 1) in all pressure extensions; i.e., the more significant the pore volume percentage, the greater the adsorption. Only IRMOF-6 does not follow this relation. The organic unit of IRMOF-6 is a better adsorption site than the organic unit of other IRMOFs; thus, the small pore volume is compensated.

On the other hand, the adsorption in IRMOF-6 (76.93% pore) is followed by IRMOF-10 (88.42% pore) at low pressures, up to 20 bar, while up to 35 bar is the IRMOF-8 (83.65% pore). After that, it approximates gradually to IRMOF-1 (80.42% pore) but never falls below, which would be expected by pore volume percentage analysis for all pressure ranges. Therefore, what is nearly relevant for gas separation is the gas-MOF intermolecular interactions becoming more attractive than the steric effect in driving adsorption in IRMOF-6 than in the others. This outcome is a characteristic of IRMOF-6, which adsorbs more efficiently than does the space it occupies. For low-pressure applications, the intermolecular interactions are relevant, which is one of the objectives of using MOFs to explore adsorption. However, IRMOF-16 will be the best if high pressures are required because it has a larger volume and consequently holds more molecules in the available volumes, while IRMOF-6 has already saturated.

To evaluate the efficiency of each structure, we use two definitions of the volumetric adsorption capacity (VAC). The VAC_c_ is defined as the number of molecules adsorbed (*N*) per unit cell divided by the volume of the respective unit cell (*V*_c_) and VAC_p_ where *N* is divided by the pore volume (*V*_p_). According to these definitions, the higher the VACs, the greater the efficiency, where both IRMOF VACs are presented in Table 2.

**Table 2:** VACs (×10^–4^ N/Å^3^) Data Concerning Unit Cell Volume (VAC_c_), and Pore Volume (VAC_p_)

	VAC_c_			VAC_p_		
MOF	1 bar	40 bar	80 bar	1 bar	40 bar	80 bar
IRMOF-1	0.65	15.49	32.38	0.80	19.26	40.27
IRMOF-6	1.40	21.85	38.16	1.81	28.40	49.60
IRMOF-8	0.63	12.96	30.10	0.75	15.49	35.99
IRMOF-10	0.57	9.64	24.15	0.64	10.91	27.31
IRMOF-14	0.75	12.58	28.95	0.81	13.67	31.45
IRMOF-16	0.33	5.80	14.86	0.36	6.36	16.27

From the data in Table 2, it is possible to verify that for all IRMOFs, the pressure increases efficiency concerning VAC_c_ and VAC_p_. IRMOF-6 provides the highest efficiency per unit of volume, which is in line with the conclusions of Yaghi et al., who, based on experimental data, chose IRMOF-6 as the most suitable for CH_4_ adsorption. The IRMOF-16, which is the one with the highest *V*_c_ and *V*_p_, was the least efficient among all, so it can be stated that the organic unit of the IRMOFs, even though it is a deficient site, plays an essential role in the adsorption efficiency and that the larger pores are not necessarily the most efficient for specific applications. Also, it is possible to observe that the characteristics of the binders, such as altering the chemical environment of the pore, are relevant at lower pressures.

**Figure 3:**
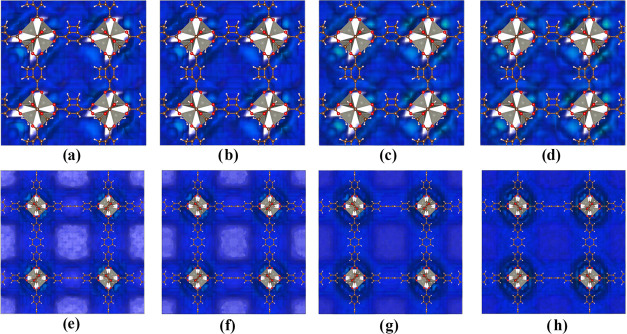
Density distribution maps for CH_4_ adsorption in IRMOF-6 (top) and IRMOF-16 (bottom), for (a) and (e) at 1 bar, (b) and (f) at 10 bar, (c) and (g) at 40 bar, and (d) and (h) at 80 bar. Density color: white: lower probability; green: greater probability.

Density distribution maps indicate that at low pressures, the CH_4_ molecules preferentially adsorb on the inorganic unit in IRMOF-16, while adsorption occurs throughout the pore in IRMOF-6. When the pressure increases, there is no significant change in the CH_4_ adsorption distribution in IRMOF-6. This result is analyzed with the pressure increase for the adsorption of CH_4_ in IRMOF-6 and IRMOF-16 (Figure 3).

As these results suggest, the two observed behaviors can be attributed to distinct forces governing these adsorptions and are key to understanding the adsorption of CO_2_ and CH_4_ on MOFs. In IRMOF-6, the attractive influence of adsorption sites extends throughout the entire pore because this structure has the smallest pore volume. Conversely, in the case of IRMOF-16, the volume available in the center of the pore is occupied only under the influence of the pressure forcing the molecules toward it.

### 3.3 Pure Component: CO_2_

The adsorption isotherms in Figure 3a demonstrated that within the evaluated pressure range (1–50 bar), IRMOF-6 has the most significant relation between masses. However, at 50 bar, the results indicate that its pore is getting saturated, unlike IRMOF-16.

**Figure 4:**
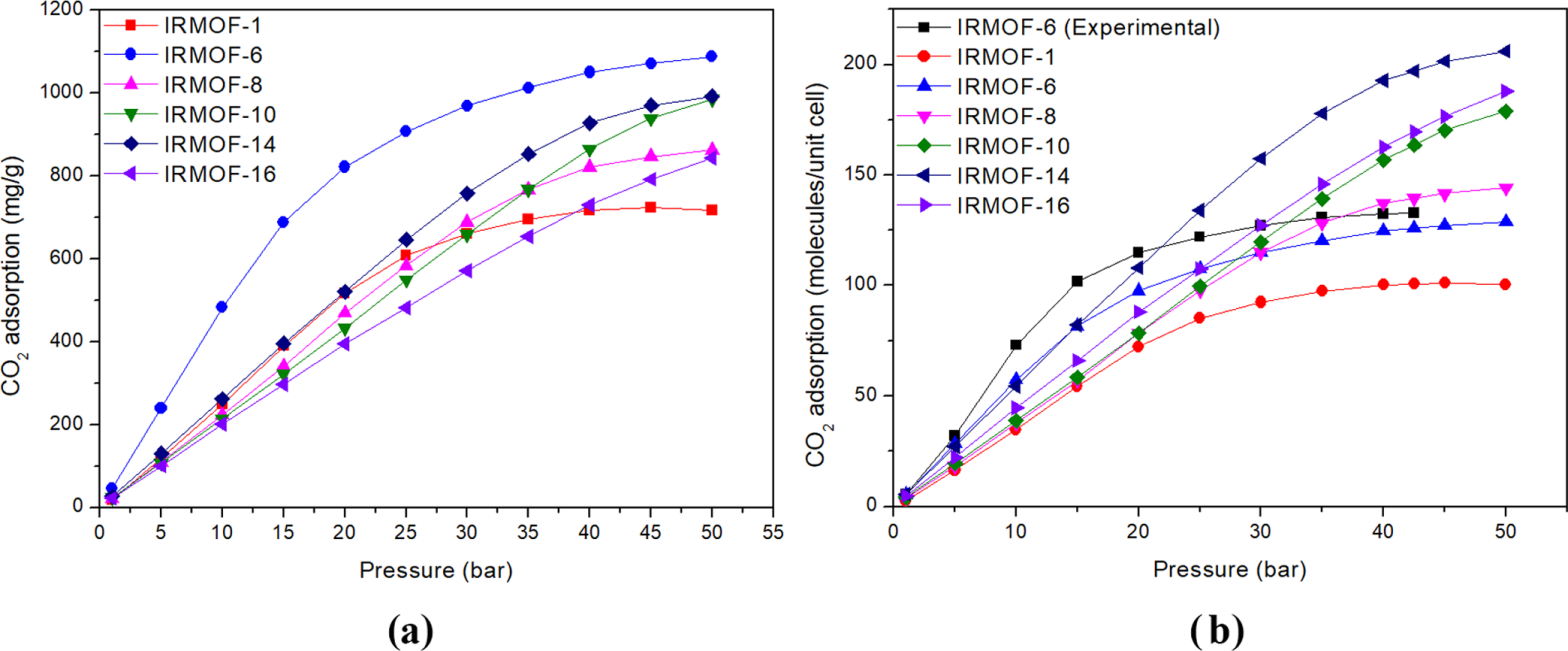
Adsorption isotherm in mg/g (a) and molecules/unit cell (b) at different pressures studied.

From the number of molecules point of view, at 10 bar, IRMOF-6 is the one that most adsorbs molecules per unit cell, but at 15 bar, IRMOF-14 becomes the one that most adsorbs, and at 50 bar, its pore appears to be saturated (around 206 molecules). Meanwhile, IRMOF-16 does not show signs of saturated as it adsorbs 188 molecules per unit cell. IRMOF-1, IRMOF-6, and IRMOF-8 are the first to have their pore saturated, as shown in Figure 4b.

Qualitatively, the CO_2_ adsorption exhibits a trend similar to that of CH_4_ adsorption, with a net zero dipole moment. Our data, which are in agreement with the experimental data measured at 298 K,^5^ provide a reliable basis for these conclusions. IRMOF 1, 8, 10, 14, and 16 follow the sequence of pore volume percentage. At the same time, IRMOF-6 displays high adsorption levels up to moderate pressures, decreasing for high pressures but consistently remaining above IRMOF-1, in opposition to pore volume percentage analysis. However, when comparing the number of adsorbed molecules per unit cell, CO_2_ adsorption on IRMOF-14 is almost 206 molecules/unit cell while CH_4_ is almost 118 molecules/unit cell at 80 bar. These results, backed by our careful computational study, further support our earlier findings at 298 K. The theoretical isotherm for IRMOF-6 proved to be in good agreement with the experimental isotherm,^54^ with good description at low and high pressures and with greater deviation for intermediate pressures, but this can be considered a good result.

The calculated VACs for the CO_2_ adsorption on the IRMOFs (Table 3) suggest an interesting trend: as the pressure increases, the efficiency of all IRMOFs also increases and the difference between them significantly decreases. IRMOF-6 is the most efficient. However, at lower pressures, the gap concerning the others is significant.

**Table 3:** VACs (×10^–4^ N/Å^3^) Data Concerning the Unit Cell Volume (VAC_c_) and Pore Volume (VAC_p_)

	VAC_c_			VAC_p_		
MOF	1 bar	20 bar	50 bar	1 bar	20 bar	50 bar
IRMOF-1	1.37	37.43	52.00	1.71	46.54	64.65
IRMOF-6	3.18	56.57	75.50	4.13	73.54	98.14
IRMOF-8	1.38	28.80	52.93	1.65	34.43	63.27
IRMOF-10	1.04	19.48	44.40	1.18	22.03	50.22
IRMOF-14	1.38	26.61	50.71	1.50	28.91	55.09
IRMOF-16	0.62	11.09	23.54	0.67	12.15	25.78

**Figure 5:**
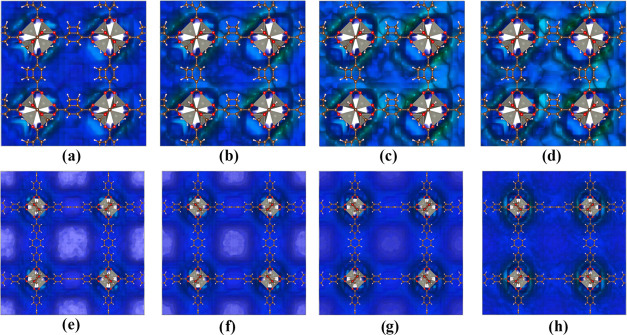
Density distribution maps for CO_2_ adsorption in IRMOF-6 (top figures) and IRMOF-16 (bottom figures), for (a) and (e) at 1 bar, (b) and (f) at 10 bar, (c) and (g) at 20 bar, and (d) and (h) at 50 bar.

The distribution density map (Figure 5) provides key insight into the adsorption process. It shows that the inorganic unit plays a pivotal role, exhibiting the most significant preference for adsorption. As the pressure increases, the zone around the inorganic unit becomes more pronounced (takes on a greener hue), indicating a higher density and reinforcing its crucial role in adsorption.

For the IRMOF-16, the pressure increase makes the regions more homogeneous, and the density around the inorganic unit also increases (Figure 5).

These maps present the same general features as those observed for CH_4_. In IRMOF-6, the molecules occupy all pore volumes, even at low pressure, whereas in IRMOF-16, the occupation of the center of the pore occurs only at high pressures. Hence, the adsorption sites of IRMOF-6 attract CO_2_ molecules all over the pore, while in the case of IRMOF-16, the CO_2_ molecules only occupy the central region of the pore at high-pressure levels.

### 3.4 Gas Mixture

**Figure 6:**
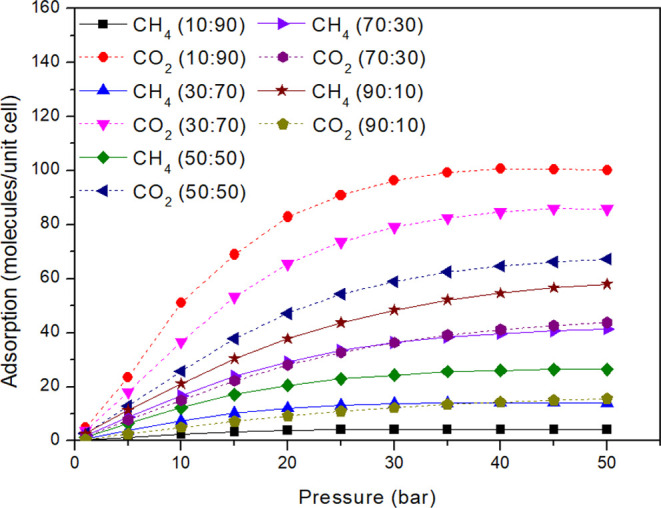
Adsorption isotherms for CH_4_ and CO_2_ mixtures in different proportions for IRMOF-6.

So far, we have investigated the behavior of systems comprising pure CH_4_ and pure CO_2_. This analysis identified that the most favorable structure to adsorb these molecules is IRMOF-6, while the less promising structure is IRMOF-16. To further understand the adsorption competition, we conducted simulations exploring the adsorption of mixtures of these two gases in IRMOF-6. Many mixture ratios, designed as CH_4_/CO_2_ (molecule ratio), were strategically considered. Specifically, the proportions were 10:90, 30:70, 50:50, 73:30, and 90:10, respectively. The isotherms produced from the simulations are listed in Figure 6.

The first case to be evaluated is for the 10:90 ratio; i.e., in the vicinity, there is a large set of molecules consisting of 10% CH_4_ and 90% CO_2_, and the molecules can migrate freely from the vicinity to the simulation box (system) or from the simulation box to the surroundings. The high concentration of CO_2_ in the vicinity facilitates access of this molecule to the pore. However, the adsorption is also influenced by the affinity of these molecules with the adsorption sites. Evaluating the CH_4_/CO_2_ ratio under saturated conditions (highest pressure), which is nearly 4:96 for 100 adsorbed molecules, we observed that the CO_2_ adsorption is higher than the mixture ratio (10:90). This indicates that the adsorption site affinity for CO_2_ is stronger than that for CH_4_. In the second case, CH_4_ is the major component (ratio of 90:10). The data contained in Figure 6 correspond to an approximate ratio of 83:17 for 100 adsorbed molecules. This ratio for CH_4_ adsorption is lower than the mixture ratio (90:10), confirming the preference of the adsorption site affinity by CO_2_, consistent with the first case. The 70:30, 30:70, and 50:50 ratios repeat the behavior, with the adsorption ratio of CO_2_ being more significant than the mixture ratio. Therefore, independent of the mixture ratio, the adsorption of CO_2_ is primarily preferred, which is in agreement with experimental studies using different porous materials.^55,56^

Another important observation is that in the 90:10 mixture for pressure, the total number of adsorbed molecules (CH_4_ + CO_2_) is around 74 molecules, 58 of CH_4_ and 16 of CO_2_. In contrast, for pure CH_4_, it is about 61 molecules. This behavior can be attributed to CH_4_ molecules interacting better with CO_2_ than CH_4_ due to the adsorption site affinity for CO_2_. The interaction energy of CH_4_–CH_4_ is 3.05 kJ/mol, for CO_2_–CO_2_ is 3.68 kJ/mol, and for CH_4_–CO_2_ is 5.52 kJ/mol. The CH_4_–CO_2_ interaction energy is the biggest, considering all gas molecules interaction in this system.

**Figure 7:**
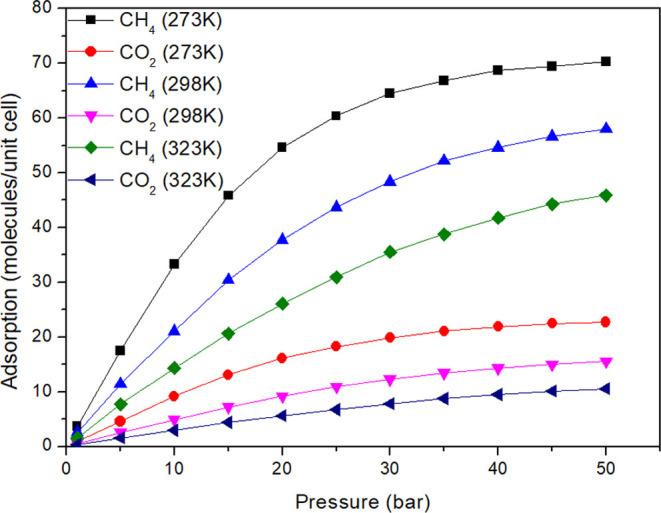
Adsorption isotherms for CH_4_ and CO_2_ mixture, in 90:10 ratio, at different temperatures.

At this time, the effect of temperature on a 90:10 mixture was investigated (Figure 7), which is the ratio analyzed closest to natural gas composition conditions. The impact of the temperature combined with the pressure variation can provide the necessary data to indicate the best conditions to maximize the separation of the studied gases. The simulation results can be seen in Figure 7.

The temperature increase causes a reduction in the adsorption of gases, as expected.^57^ This reduction happens due to the increase in the system internal energy and, consequently, the breaking of weak bonds. The final balance is a reduction in the number of adsorbed molecules. The results also indicate that the temperature reduction increases the difference between the number of adsorbed molecules of CH_4_ and CO_2_. Considering 50 bar of pressure, the difference between the adsorbed CO_2_ and CH_4_ molecules is approximately 35 molecules of CO_2_ at 323 K. At 298 K, this difference is close to 42 molecules of CO_2_, and at 273 K, it increases to next to 48 molecules.

Table 4 shows the percentage of molecules in the 90:10 mixture. Reducing the temperature increases the adsorption of CO_2_ more effectively. For the temperature of 273 K, which is the most effective, at 1 bar, the MOF adsorbs approximately four molecules of CO_2_ against one molecule of CH_4_, and then 79.78% of the adsorbed molecules are CH_4_. For pressure equal to 25 bar, 60 molecules of CH_4_ are adsorbed against 18 molecules of CO_2_, which means that 76.77% of the adsorbed molecules are CH_4_. In contrast, when the pressure rises to 50 bar, 70 molecules of CH_4_ adsorbed against 22 of CO_2_, indicating that 75.57% of the adsorbed molecules are CH_4_. Values for other pressures can be seen in Table 4.

**Table 4:** Percentage of Molecules of 90:10 Mixture (CH_4_/CO_2_) Adsorbed on IRMOF-6 at Different Pressures for a Temperature of 273 K

	composition (%)					
	273 K		298 K		323 K	
pressure (bar)	CH_4_	CO_2_	CH_4_	CO_2_	CH_4_	CO_2_
1	79.78	20.22	81.57	18.43	83.27	16.73
5	79.23	20.77	81.55	18.45	82.96	17.04
10	78.41	21.59	81.02	18.98	82.72	17.28
15	77.77	22.23	80.83	19.17	82.40	17.60
20	77.21	22.79	80.47	19.53	82.22	17.78
25	76.77	23.23	79.96	20.04	82.03	17.97
30	76.46	23.54	79.75	20.25	81.93	18.07
35	76.04	23.96	79.49	20.51	81.57	18.43
40	75.86	24.14	79.19	20.81	81.53	18.47
45	75.56	24.44	79.06	20.94	81.37	18.63
50	75.57	24.43	78.85	21.15	81.25	18.75

The data in Table 4 provide an essential insight: increasing the pressure reduces the percentage of adsorbed CH_4_ molecules and increases the rate of adsorbed CO_2_. This behavior suggests that a high pressure and low temperature are optimal conditions for separating these gases. However, it is important to note that even under these conditions, the separation efficiency is low, highlighting the need to investigate the nature of interactions.

### 3.5 Nature of Interactions

It is important to study the system interactions and characteristics to gain deeper insight into the results. This analysis was performed in two steps. First, we will compare the adsorption enthalpy^58^ obtained by GCMC with data from the literature (Table 5). Then, we evaluated the enthalpy for each gas in each IRMOF at 298 K and pressures of 1 and 50 bar (Table 6).

**Table 5:** Adsorption Enthalpy for Pure Gases^a^

	adsorption enthalpy (kJ/mol)			
MOF	CH_4_	CO_2_	*T* (K); *P* (bar)	references
IRMOF-1	–12.43 (−12.30)		298; 65	(59)
IRMOF-1		–13.02 (−14.90)	353; 1.33	(60)
IRMOF-14	–9.58 (−10.00)		298; 35	(61)

a Literature data in parenthesis.

**Table 6:** Adsorption Enthalpy at 298 K, 1 and 50 bar, for CH_4_ and CO_2_ in IRMOFs

	adsorption enthalpy (kJ/mol)			
	1 bar		50 bar	
MOF	CH_4_	CO_2_	CH_4_	CO_2_
IRMOF-1	–9.98	–13.12	–12.08	–20.35
IRMOF-6	–11.43	–14.92	–14.18	–22.71
IRMOF-8	–9.24	–12.64	–10.36	–17.92
IRMOF-10	–8.75	–11.85	–9.58	–15.26
IRMOF-14	–9.38	–12.70	–9.88	–16.67
IRMOF-16	–7.21	–10.34	–7.34	–11.58

When the adsorption enthalpy values found in the literature are compared with the values calculated using the methodology of this work, it is possible to verify an excellent agreement. The calculated CH_4_ adsorption enthalpies in IRMOF-1 and IRMOF-14 show a difference of only 1.05 and 4.20%, respectively, from the literature. For CO_2_ in IRMOF-1, a more pronounced deviation is observed, presenting a difference of 12.62%.

When the adsorption enthalpy for each gas in each IRMOF is evaluated at 298 K and pressures of 1 and 50 bar, two essential points can be highlighted in this analysis. One of them is that adsorption in IRMOF-6 generates a system with lower energy for both gases, with CO_2_ being the most favorable in accordance with the literature.^62^ The other point is that the pressure increase causes a decrease in the adsorption enthalpy, which is more accentuated for CO_2_. Therefore, CO_2_ has a more significant adsorption advantage when the pressure is high in the competition between the two gases.

**Figure 8:**
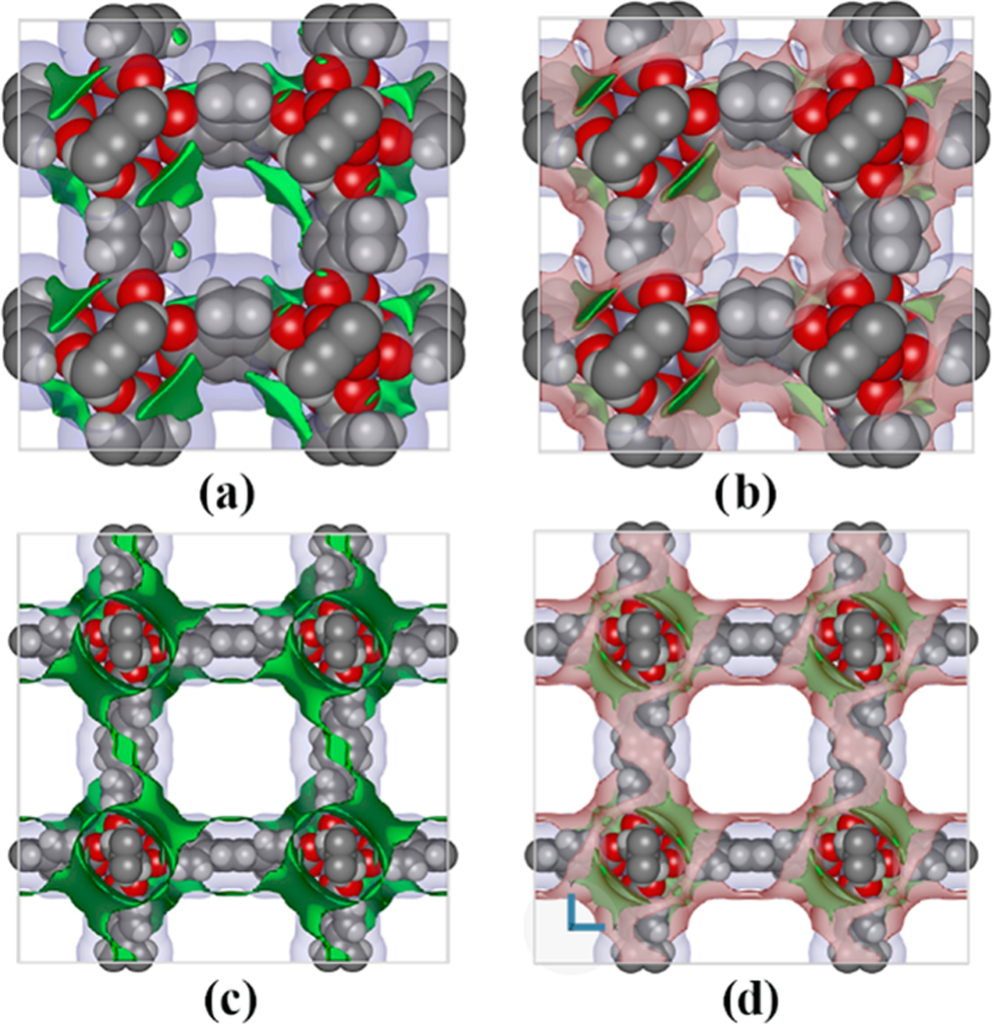
Energy surfaces for (a) CH_4_ in IRMOF-6 with isocontour values of 0 kJ/mol (blue) and −11.43 kJ/mol (green), (b) CO_2_ in IRMOF-6 with isocontour values of 0 kJ/mol (blue), −11.43 kJ/mol (red), and −14.92 kJ/mol (green), (c) CH_4_ in IRMOF-16 with isocontour values of 0 kJ/mol (blue), and −7.21 kJ/mol (green), and (d) CO_2_ in IRMOF-16 with isocontour values of 0 kJ/mol (blue), −7.21 kJ/mol (red), and −10.34 kJ/mol (green).

Taking as a reference the adsorption enthalpy values for CH_4_ and CO_2_ in IRMOF-6 and IRMOF-16 at 1 bar and 298 K, it was possible to better understand which regions are preferred in each energy range, as seen in Figure 8.

For IRMOF-6, it is possible to observe that for an interaction energy range exceeding 11.43 kJ/mol, CH_4_ molecules preferentially adsorb in regions close to the inorganic unit (green region in Figure 8a). In contrast, for CO_2_ in the same energy range, it is observed that the encompassing region is much larger (region in red in Figure 8b). Therefore, in a dispute between the studied molecules, CO_2_ has a higher adsorption probability compared to CH_4_. This advantage continues with energies greater than 14.92 kJ/mol, where CO_2_ molecules can adsorb (green region in Figure 8b). Our results of Table 6 are in accordance with the GCMC that showed an isosteric heat of adsorption of about 14.38 kJ/mol for CO_2_ and 10.31 kJ/mol for CH_4_.^62^ Furthermore, CO_2_ adsorption energies are more strength than CH_4_ at all pressures, and CO_2_ has the wider energy surface distributed in IRMOF-6 and IRMOF-16.

For IRMOF-16, different behavior is observed in the energy range of up to 7.21 kJ/mol. CH_4_ and CO_2_ can adsorb in very similar proportions. Hence, a greater competition between CH_4_ and CO_2_ (green region in Figure 8c and red region of Figure 8d, respectively) is expected. With decreasing energy, the molecules begin to adsorb increasingly closer to the inorganic unit, and with energy greater than 10.34 kJ/mol, only CO_2_ is adsorbed, making a significant shift in the adsorption.

## 4 Conclusions

Our simulations with the gases individually revealed that IRMOF-6 exhibits superior efficiency when considering the pore and unitary cell volumes. Conversely, IRMOF-16 demonstrated the least efficiency. This trend was consistent for both gases, aligning with existing literature on CH_4_. Our study of gas mixtures in varying proportions and subsequent comparison of these proportions shed light on the number of molecules adsorbed on IRMOF-6 and the competition between the gases. It became evident that the adsorption of CO_2_ molecules is favored, albeit not significantly. However, a notable observation was that as the temperature decreases, the competition tilts in favor of CO_2_ adsorption, making IRMOF-6 a promising candidate among the IRMOFs under evaluation. Adsorption enthalpy data and isocontour values provided a better understanding of the most attractive adsorption regions for each gas and the reasons why CO_2_ adsorption is more efficient. Its adsorption produces a more expressive reduction in system energy.

## Data Availability

All data and information for the reproduction of these works are available in the text.

## Supporting Information Available

Table with the Lennard-Jones parameters from GenericMOFs force field and used to model IRMOFs, CO_2_, and CH_4_ (PDF)
