# Supplementary material for: Theoretical Study of CH4 and CO2 Separation by IRMOFs
Source: ACS Omega. 2024 Sep 4;9(37):38686–95. doi: 10.1021/acsomega.4c04482 (PMC11411654; doi:10.1021/acsomega.4c04482)
Supplement: Supplementary file 1 — ao4c04482_si_001.pdf [file ao4c04482_si_001.pdf]

Supporting Information

Theoretical study of CH_4 and CO_2 separation by IRMOFs

Ana Luiza Andrade Mizuno[1], Edna da Silva Machado[1], João B. L. Martins[1], José Roberto dos Santos Politi[1], and Nailton Martins Rodrigues[1,2]*

[1]*Instituto de Química, Universidade de Brasília, 70910-900, Brasília - DF, Brasil.*

[2]*Departamento de Química, Universidade Federal do Maranhão, 65085-580, São Luís - MA, Brasil.*

Table S1. The Lennard-Jones parameters from GenericMOFs force field and used to model IRMOFs, CO_2 and CH_4.

**Component**	**Pseudo atom**	σ **(Å)**	ε/k_b **(K)**
IRMOFs	Zn	2.46155	62.3992
	O_cen	3.03315	48.1581
	O_CO2	3.05000	79.0000
	C_CO2	2.80000	27.0000
	C_benz	3.60000	30.7000
	H	2.36000	25.4500
CO_2	O_CO2	3.05000	79.0000
	C_CO2	2.80000	27.0000
CH_4	CH4_sp3	3.64000	148.0000

The ChelpG charge for CO_2 molecule: C = 0.496726e^- and O = -248363e^-.
